# Comparison of hierarchical clustering and neural network clustering: an analysis on precision dominance

**DOI:** 10.1038/s41598-023-32790-3

**Published:** 2023-04-06

**Authors:** Nazish Shahid

**Affiliations:** Department of Mathematics, Forman Christian College (A Chartered University), Lahore, Pakistan

**Keywords:** Environmental sciences, Ocean sciences, Mathematics and computing

## Abstract

A comparison of neural network clustering (NNC) and hierarchical clustering (HC) is conducted to assess computing dominance of two machine learning (ML) methods for classifying a populous data of large number of variables into clusters. An accurate clustering disposition is imperative to investigate assembly-influence of predictors on a system over a course of time. Moreover, categorically designated representation of variables can assist in scaling down a wide data without loss of essential system knowledge. For NNC, a self-organizing map (SOM)-training was used on a local aqua system to learn distribution and topology of variables in an input space. Ternary features of SOM; sample hits, neighbouring weight distances and weight planes were investigated to institute an optical inference of system’s structural attributes. For HC, constitutional partitioning of the data was executed through a coupled dissimilarity-linkage matrix operation. The validation of this approach was established through a higher value of cophenetic coefficient. Additionally, an HC-feature of stem-division was used to determine cluster boundaries. SOM visuals reported two locations’ samples for remarkable concentration analogy and presence of 4 extremely out of range concentration parameter from among 16 samples. NNC analysis also demonstrated that singular conduct of 18 independent components over a period of time can be comparably inquired through aggregate influence of 6 clusters containing these components. However, a precise number of 7 clusters was retrieved through HC analysis for segmentation of the system. Composing elements of each cluster were also distinctly provided. It is concluded that simultaneous categorization of system’s predictors (water components) and inputs (locations) through NNC and HC is valid to the precision probability of 0.8, as compared to data segmentation conducted with either of the methods exclusively. It is also established that cluster genesis through combined HC’s linkage and dissimilarity algorithms and NNC is more reliable than individual optical assessment of NNC, where varying a map size in SOM will alter the association of inputs’ weights to neurons, providing a new consolidation of clusters.

## Introduction

Clustering is the process of assembling similar characteristic objects or variables in groups, called clusters. Due to significance of clustering applications in data analysis, pattern recognition, image processing, information retrieval and medical imaging, it has been widely studied^1–4^ using legion of computing methods. A sound clustering algorithm efficiently undertakes the tasks of scaling a non-uniform data, analyzing a categorical, numerical and binary data, interpreting the results comprehensively, revealing significant features of a system and handling a high dimensional data. The most commonly used clustering methods are Hierarchical Clustering and K-Means Clustering^5–16^. In Hierarchical Clustering, a hierarchy of clusters is built, where each cluster contains similar characteristics data points of a system. In K-Means Clustering, clusters are generated through averaging the data by means of K centroids. Zhao et al.^17^ Asserted that consistency of hierarchical clustering solutions at different levels of granularity allowed flat partitions of disparate granularity, making them ideal for interactive exploration and visualization. For the case where clusters have many sub-clusters, hierarchical structure was deemed a natural constrain on the underlying application domain (biological taxonomy, phylogenetic trees)^18^. In an effort to seek efficient algorithms of clustering to describe the learned representation, required for features detection, Neural Network Clustering (NNC) has been preferred lately^19–26^. Du^27^ underlined the importance of self-organizing map (SOM) as one of the competitive learning based clustering neural networks to retrieve wealth of information from huge databases or the world wide web (WWW). It was narrated that structural features needed to be detected first in a database for data mining, and exploratory technique of self-organization seemed particularly promising. Jain et al.^28^ presented database segmentation, predictive modeling, and visualization of large databases as some of clustering approaches for data mining. It was maintained by^29^ that web mining was a difficult process due to undefined features of less structured WWW database. In this regard, the topology-preserving feature of SOM-NNC made it particularly suitable for web-information processing^27^.

Zhang et al.^16^ exhibited that hierarchical dendrograms in consolidation with heat maps provided sharp visualization of clinical research with heterogeneous study population. A Cognitive Comparison-Enhanced Hierarchical Clustering (CCEHC) system was proposed by^30^ to provide personalised product recommendations based on user preferences. It was emphasized that HC along with cognitive comparison-enhanced measure can improve the accuracy of cluster bounds, encompassing data elements of similar features. Cirrincione et al.^31^ proposed that the drawback of splitting threshold setting by HC can be addressed by NNC by building a hierarchical tree in an incremental and self-organized way. Based on the novel idea of neighborhood convex hull, this technique defines horizontal growth by means of an anisotropic region of influence. Furthermore, the synthesis of hierarchical segmentation and GH-EXIN neural-network was deemed to improve the accuracy of clustering. Okamoto et al.^32^ illustrated that at each node of a hierarchical classification tree, log-linearized Gaussian mixture networks could be utilized as classifiers to divide the data into two subclasses based on statistical information, which are then classified into secondary subclasses and so on. It was further specified that training technique of cross validation could be adopted to prune unnecessary structure of a cluster tree. A neural network, Self-Organizing Tree Algorithm (SOTA) was used by^33^ for the analysis of gene expression data. It was demonstrated that the result of the algorithm was a hierarchical cluster obtained with accuracy and robustness of a neural network. Moreover, it was clarified that SOTA clustering had an advantage over classical hierarchical clustering, where clustering process is conducted from top to bottom and the highest hierarchical levels are resolved before going to the details of the lowest levels. However, the growing can be stopped at the desired hierarchical level with SOTA using the criterion based on an approximate distribution of probability, obtained by randomisation of original data set. To take this investigation to a further level, we have defined the stopping criteria of cluster trees with the help of ML-stem algorithm.

Mangiameli et al.^34^ conducted a comparison of SOM-NNC and HC, and demonstrated the superiority of NNC over HC. It was presented that HC methods had tendency to commit classification errors when empirical data departed from ideal conditions of compact isolated clusters. However, the superiority of NNC over HC resolutely depended on attaining precision accuracy of decisions through NNC on a partially structured data. It can be noted that^31–34^ emphasized on improvement of clustering accuracy through an NNC-HC association. In the current investigation, we examined Hierarchical Clustering conduct of a normalized water concentration data through ML-clusterdata algorithm. Dual clustering efficiency was sought for HC by association of dissimilarity matrix and linkage matrix. In comparison, we obtained clustering information of water variables through visual analysis of SOM-NNC features. The study of SOM output maps not only revealed clustering structure formed of data predictors, it also highlighted atypical features of the input data. Additionally, we have endeavored to seek an efficient algorithm to determine clustering boundaries of water ensemble by using NNC and HC.

## Materials and methods

### Study region

In order to study water characteristics, several locations of Lahore, the capital of Punjab province of Pakistan (located between $31^{\circ }15^{\prime }-31^{\circ }45^{\prime }\, \textrm{N}$ and $74^{\circ } 01^{\prime }-74^{\circ }39^{'}\,\textrm{E}$), were chosen and samples were collected. Lahore, the second largest city of Pakistan, covers a total land area of 1772 ${\text {km}} ^{2}$.

### Monitoring locations and samples collection protocol

Pakistan council of research in water resources (PCRWR) under its flagship program National Level Water Quality Monitoring 2020 had established a monitoring station in Regional Office Lahore. In consideration of water usage points, storage centres, filter plants, water sources disposition, grid structure and distributing convenience, 16 central cites were elected for the purpose of sample collection in a grid size of 16 $\hbox {km}^2$. In accordance with APHA (American Public Health Association) 2017 protocols, four types of samples were collected from each site, and were tested in Lahore examination station. These four types^35^ were classified as A, B, C and D for the purpose of microbiological, trace elements, nitrate and physio-chemical parameters testing, respectively. Elementary protocols in advance of types B, C and D testing included rinsing of bottles with deionized water before addition of preservatives and transportation of samples for chemical analysis without iceboxes. The transportation of type A samples was conducted in disinfected and insulated lightproof packaging of controlled temperature range 2–$8\,^\circ$C. An immediate testing was ensured for residual chlorine, pH and turbidity to prevent them from changing the colour during storage and transportation. In keeping with APHA 2017 protocols, 6-hour time period was maintained between collection of a sample and its testing, and the water was allowed to flow for at least ten minutes before its collection for an accurate source representation.

### Data components

**Figure 1:**
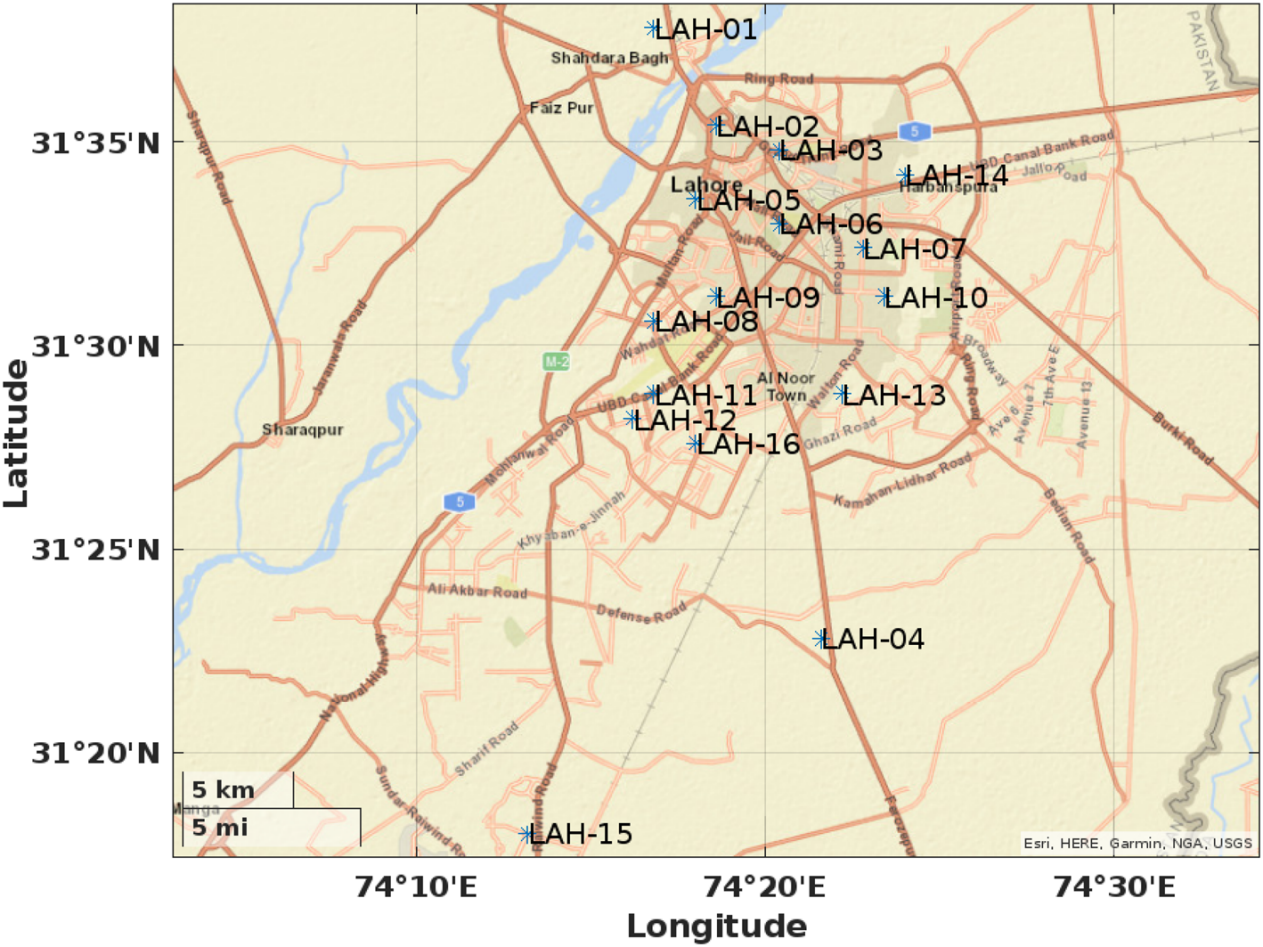
Coordinates placement of elected locations in lahore (https://www.mathworks.com/help/matlab/ref/geobasemap.html).

The data used for current analysis is comprised of water elements’ concentration in samples collected in year 2020^35^ from 16 elected locations of Lahore. These locations are assigned specific codes, and are exhibited on a map^36^, Fig. 1 according to their coordinates’ placement. To conduct a comparative investigation, concentration values of water parameters, Electrical Conductivity (EC), pH, Turbidity, Bicarbonate ($\text {HCO}_3$), Carbonate ($\text {CO}_3$), Calcium (Ca), Magnesium (Mg), Hardness (Hard), Chloride (Cl), Sodium (Na), Potassium (K), Sulphate (${\text {SO}}_4$), Nitrate (${\text {NO}}_3$), TDS, Iron (Fe), Fluoride (F), Arsenic (As), Total Coliforms and *E. Coli* are considered. A catalogue of parameters’ measuring units and diagnostic techniques used for aggregate evaluation is presented in Table 1.

**Table 1:** Water parameters, their measuring units and diagnostic techniques.

Parameters	Abbreviation	Units	Diagnostic techniques
Electrical Conductivity	$\text {EC}$	$\mu \hbox {S/cm}$	$\text {EC Meter Hach-44600-00 USA}$
pH	$\text {pH}$	$\text {pH unit}$	$\text {pH Meter Hanna Instrument, Model 8519 Italy}$
Turbidity	$\text {Turbidity}$	$\text {NTU}$	$\text {Turbidity Meter Lamotte, Model 2008 USA}$
Bicarbonate	${\text {HCO}}_{3}$	$\text {mg/l}$	$\text {2320 Standard Method APHA 2017}$
Carbonate	${\textrm{CO}}_{3}$	$\text {mg/l}$	$\text {2320 Standard Method APHA 2017}$
Calcium	$\text {Ca}$	$\text {mg/l}$	$\text {3500-Ca-D Standard Method APHA 2017}$
Magnesium	$\text {Mg}$	$\text {mg/l}$	$\text {2340-C Standard Method APHA 2017}$
Hardness	$\text {Hard}$	$\text {mg/l}$	$\text {EDTA Titration Standard Method APHA 2017}$
Chloride	$\text {Cl}$	$\text {mg/l}$	$\text {Titration Standard Method APHA 2017}$
Sodium	$\text {Na}$	$\text {mg/l}$	$\text {Flame Photometer PFP7 UK}$
Potassium	$\text {K}$	$\text {mg/l}$	$\text {Flame Photometer PFP7 UK}$
Sulphate	${\textrm{SO}}_{4}$	$\text {mg/l}$	$\text {SulfaVer4 Hach-8051 by Spectrophotometer}$
Nitrate	${\textrm{NO}}_{3}$	$\text {mg/l}$	$\text {Cd. Reduction Hach-8171 by Spectrophotometer}$
TDS	$\text {TDS}$	$\text {mg/l}$	$\text {2540C Standard Method APHA 2017}$
Iron	$\text {Fe}$	$\text {mg/l}$	$\text {Spectrophotometer Standard Method APHA 2017}$
Fluoride	$\text {F}$	$\text {mg/l}$	$\text {4500-FC Ion-Selective Electrode Standard Method APHA 2017}$
Arsenic	$\text {As}$	$\mu \hbox {g/l}$	$\text {AAS Vario 6, Analytik Jena AG 3111B APHA 2017}$
Total Coliforms	$\text {Total Coliforms}$	$\text {CFU/100 ml}$	$\text {9221-B, C } \& \text { D, Standard Method APHA 2017}$
*E. Coli*	*E. Coli*	$\text {CFU/100 ml}$	$\text {9221-B, C } \& \text { D, Standard Method APHA 2017}$

## Statistical methods’ application

To determine an improved machine learning approach presenting an optimised analysis of pattern based results from a large data, two methods, Neural Network Clustering (NNC) and Hierarchical Clustering (HC), are employed. The features highlighted by both methods are assessed to demonstrate dominance of either of the schemes in proposing an accuracy precision.

### Neural network clustering (NNC)

To partition the data into clusters and to reduce its dimensionality, a method of grouping the predictors by similarity is administered through neural network clustering.

#### Neural network constitution

A network of self-organizing feature map (SOFM)/self-organizing map (SOM) is elected to cluster water variables. This map learns to classify variables according to how they are grouped in an input space. An SOM conducts its training on differing variables occupying competitive layers of neighbouring neurons by learning to recognise neighbouring sections of the input space. Thus, both the distribution and topology of input variables (vectors) are learnt through SOM.

#### Batch algorithm method

A version of SOFM training, called the batch algorithm, presents the whole data set to network before any weights are updated. The algorithm then determines a winning neuron for each input vector. Each weight vector then moves to the average position of all of the input vectors for which it is a winner, or for which it is in the neighbourhood of a winner.

#### Data design

We consider water parameters (Table 1), defining the water stature of a location, as predictors and locations from where these water samples have been collected as input vectors. Specifically, there are 16 input vectors and 18 water parameters.

#### Training of SOM network

**Figure 2:**
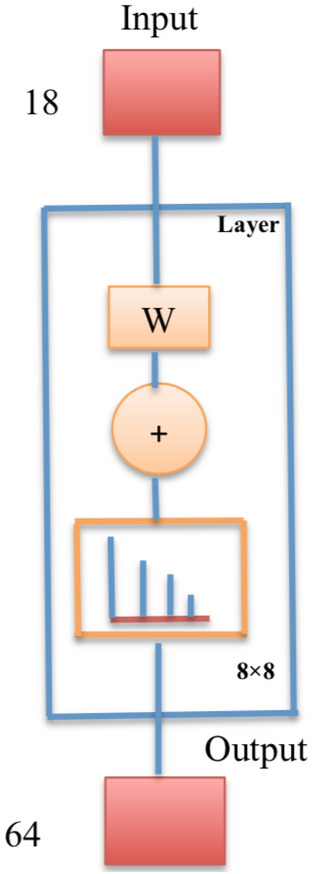
Training network.

**Figure 3:**
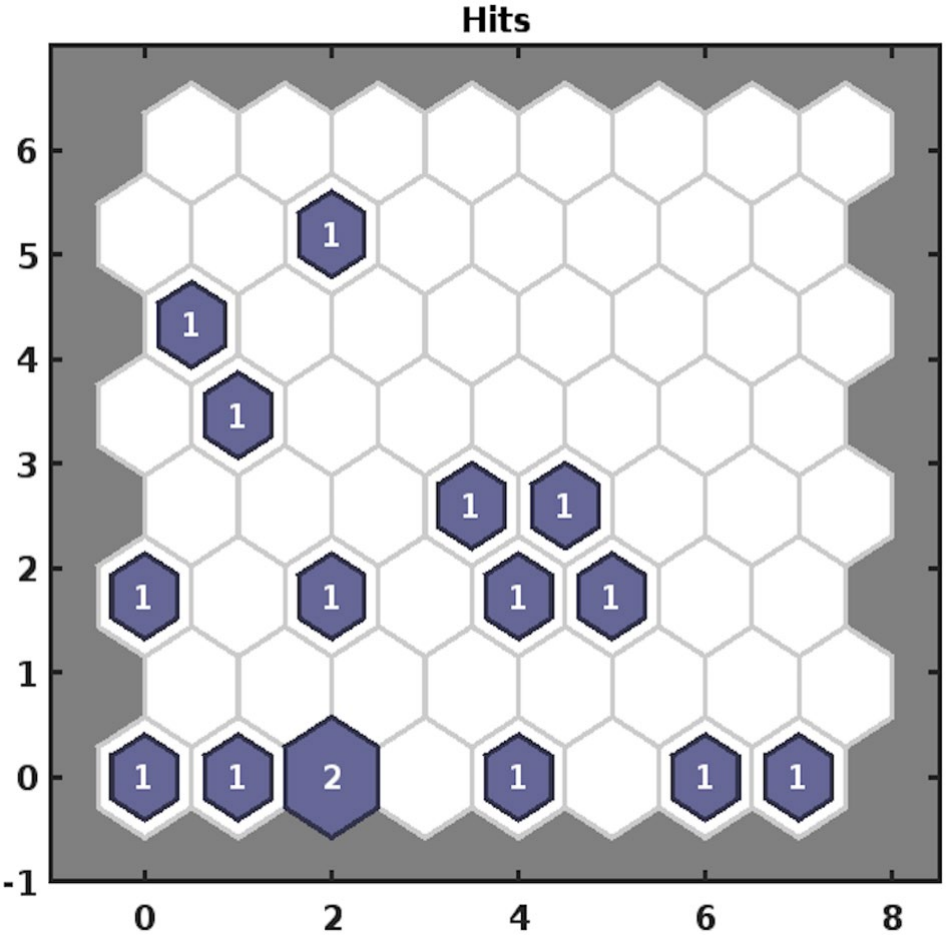
Sample hits plot.

A network of 8-size map Fig. 2 was trained until the stopping criteria of 200 epochs was reached. The map size corresponds to the number of rows and columns in the grid Fig. 3. The total number of neurons (weights associated to the variables) will be total number of points in the grid i.e. $8 \times 8=64$. In Fig. 2, an 18-predictor variables data is fed to SOM network, where neurons become layered and congregated due to characteristic similarities. Finally, SOM clusters the related neurons in a 64-grid map.

##### SOM sample hits

To assess how many input vectors are associated with each neuron, we obtain an SOM sample hits plot Fig. 3. The number on a shaded neuron represents its alliance with an input vector. This visualisation shows that maximum number of input vectors associated with a neuron is 2. Moreover, the group proximity of some neurons implies packaging of 12 input vectors into 4 clusters, and the remaining 4 input vectors linked with 4 disconnected neurons can be placed in 4 distinct clusters.

##### SOM neighbour weight distances

**Figure 4:**
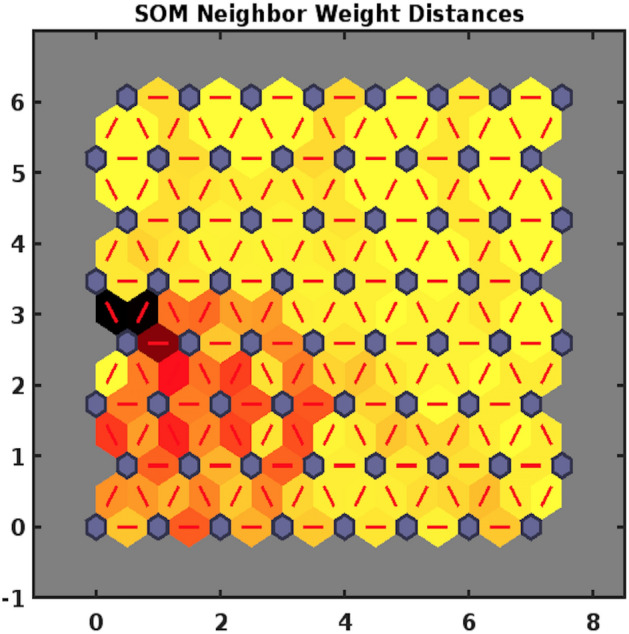
Weight distances plot.

To visualise all weights in an 18-dimensional input space (there are 18 water parameters for each input vector), we obtain an SOM neighbour weight distances plot Fig. 4. The characteristics of this plot are shown in an $8\times 8$ hexagonal grid, called SOM topology, where each hexagon represents a neuron. During the training process, weight vector associated with each neuron moves to become centre of a cluster of input vectors. The plot attributes are explained through colour-coding. The neurons (cluster centres/weight vectors) are represented through blue hexagons; red lines connect the neighbouring neurons; distances between neurons are indicated through colours in the regions containing the red lines; larger distances are represented through darker colours and smaller distances are represented through lighter colors.

It can be observed from Fig. 4 that only a quarter of the data shows dissimilarity among input vectors based on larger distances between neurons’ weight vectors corresponding to dark coloured patches. It is indicated that in this section only a small number of neurons are connected through a black patch, demonstrating strong dissimilarity between very few input vectors. The remaining three-quarter of the plot mostly connects neurons through yellow patches implying smaller distances between them, hence lesser disparity among 75% of the input vectors. These features render significant information that only a few among 16 elected locations suggest the presence of one or more outlier elements or atypical feature.

##### SOM input planes

In order to visualise weights (related to input vectors’ elements), we obtain a weight plane figure. A weight plane is configured corresponding to each element of an input vector (there are 16 input vectors, each having an 18 number of elements). It visualises the weights connecting each input to each of the neurons. Larger and smaller weights in the plot are represented by lighter and darker colours, respectively. The similar connection pattern of input elements demonstrates that those elements are highly correlated.

**Figure 5:**
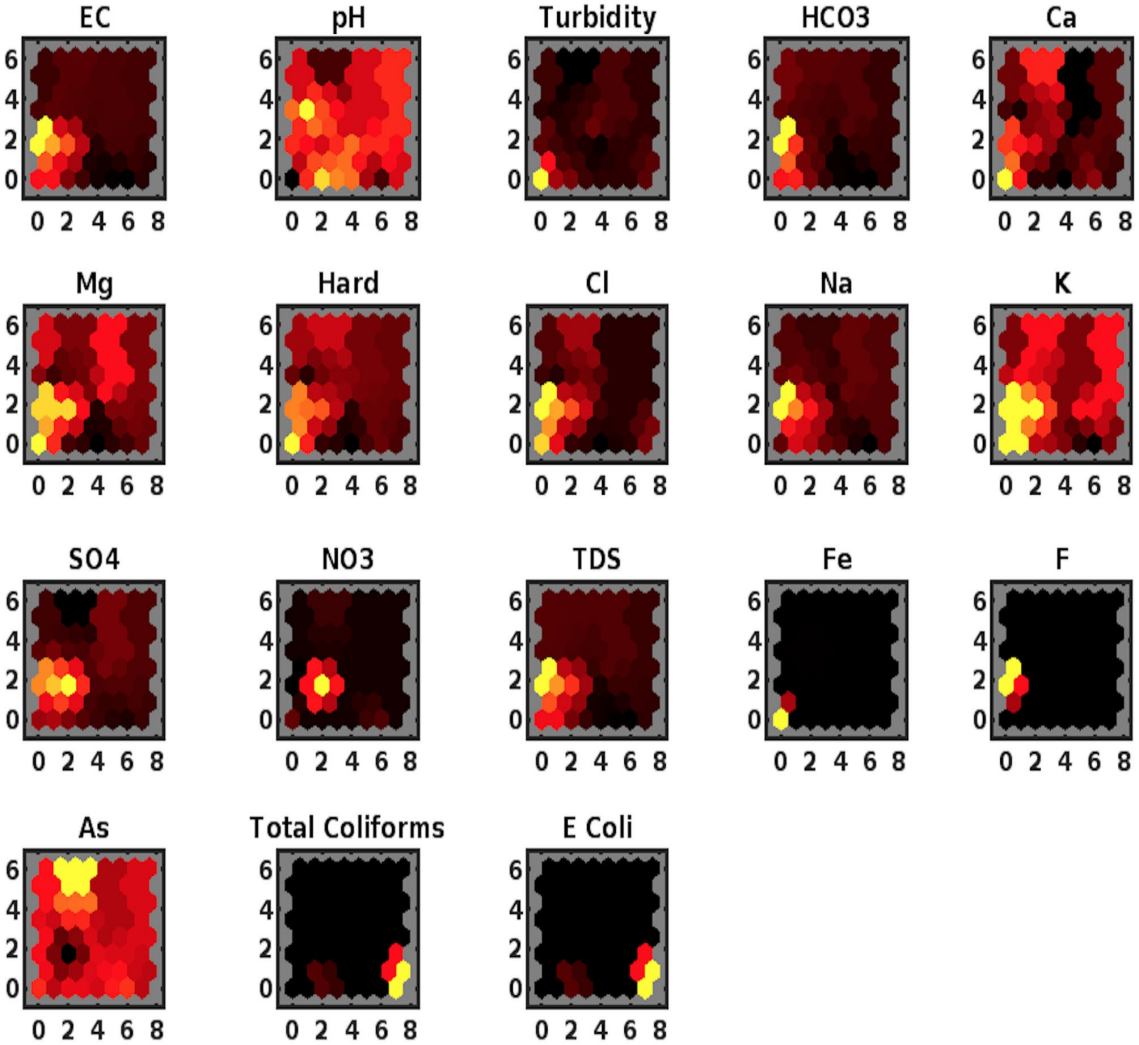
Weight planes plot.

Figure 5 exhibits subplots for input elements EC, pH, Turbidity, $\text {HCO}_3$, Ca, Mg, Hard, Cl, Na, K, ${\text {SO}}_4$, ${\text {NO}}_3$, TDS, Fe, F, As, Total Coliforms and *E. Coli*. In each plot, the connection of weights corresponding to a particular input with the layer’s neurons is represented by three prominent colours. The yellow, red and black colours demonstrate the most positive connections, no connection and the most negative connections, respectively. Some observations from Fig. 5 are highlighted as

- Based on the position of two yellow neurons on left in 4th quadrant, the inputs EC, $\text {HCO}_3$, Cl, Na, TDS and F are likely to be clustered together.
- Viewing the position of a single yellow neuron in 4th quadrant, in the bottom on extreme left, the inputs Turbidity, Ca, Hard and Fe can be placed in a single cluster based on similar strongest positive connections. However, Fe weight plane contrasts to the others due to its mostly negative connections, exhibited through black shaded neurons.
- Observing the weight planes of inputs Mg and K, a small cluster of yellow neurons in 4th quadrant shows larger weights associated to these neurons. In view of similar pattern of strongest positive connections, these two variables can be placed in a distinct cluster.
- A keen examination of weight planes of ${\text {SO}}_4$ and ${\text {NO}}_3$ illustrates a yellow neuron in the middle of 4th quadrant surrounded by red/light red neurons. Moreover, these planes display the remaining three quadrants occupied by red/black neurons, reflecting on similar negative connections’ pattern. In view of these similarities, the inputs ${\text {SO}}_4$ and ${\text {NO}}_3$ can be slotted in a distinct cluster.
- An excessive degree of resemblance between the weight planes of Coliforms and *E. Coli* is reflected through analogy of the most positive (yellow), the most negative (black) and no (red) connections between the input weights and neurons.
- It is indicated through the weight planes of pH and As that despite the difference of yellow neurons’ position in both plots, these inputs demonstrate minimum (black) or no (red) connections to the neurons here. Taking into account of this conformity, pH and As can be placed into two different clusters.

### Hierarchical clustering (HC)

To characterize the variables showing similar attributes in a water concentration data, a method of hierarchical clustering^37,38^ is used. The allocation of variables in clusters allows to determine integrated movement or influence of specific clusters on a system. Clustering was conducted on water components in Table 2.

**Table 2:** Numbers assigned to variables for cluster analysis.

Variable	EC	pH	Turbidity	$\text {HCO}_{3}$	Ca	Mg	Hard	Cl	Na	K	$\text {SO}_{4}$	$\text {NO}_{3}$	TDS	Fe	F	As	TotalColi	*E. Coli*
Assigned Number	1	2	3	4	5	6	7	8	9	10	11	12	13	14	15	16	17	18

**Figure 6:**
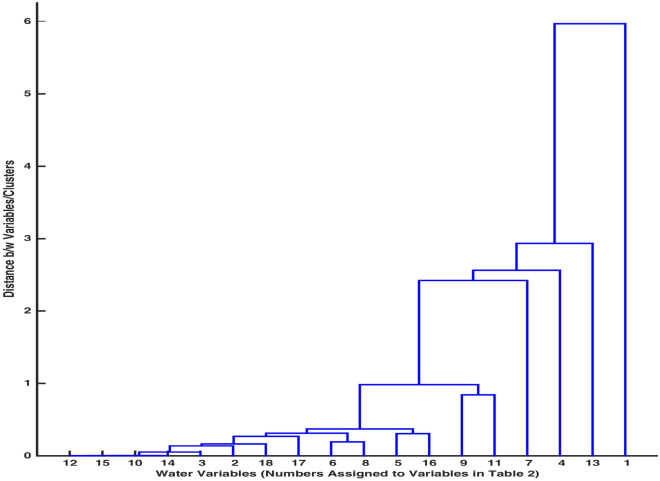
Dendrogram for clusters.

To assemble water components in groups, a cluster tree was obtained using an ML program ‘clusterdata’. A cluster tree represents stacks of clusters on different levels, each level containing variables showing an allied tendency to affect the whole system. The application of clusterdata on normalized values of 18-variables data assisted in configuration of dendrogram, Fig. 6, using dissimilarity matrix and linkage matrix. The dissimilarity matrix presented distance between every pair of variables, and the linkage matrix provided a link between every two variables or clusters. In addition to linking variables and clusters, the linkage function computed distance between a pair of variables or a pair of clusters or a cluster and a variable. The coupling of variables and clusters has been demonstrated in Table 3, where ‘0.00’ in the third row indicates the closest proximity distance between two variables/a variable and a cluster.

**Table 3:** Distance between two variables/clusters.

Variable 1	12 (${\text {NO}}_3$)	10 (K)	14 (Fe)	3 (Turbidity)	2 (pH)	18 (*E. Coli*)	6 (Mg)	17 (Total Coliforms)	5 (Ca)
Variable 2	15 (F)	19	20	21	22	23	8 (Cl)	24	16 (As)
Distance	0.00	0.00	0.00	0.05	0.14	0.17	0.19	0.27	0.31
Variable 1	25	27	9 (Na)	29	7 (Hard)	4 ($\text {HCO}_3$)	13 (TDS)	1 (EC)	
Variable 2	26	28	11 (${\text {SO}}_4$)	30	31	32	33	34	
Distance	0.31	0.37	0.84	0.98	2.42	2.56	2.93	5.97	

To validate the creation and linking of clusters in Fig. 6, cophenetic coefficient; measuring the correlation between distance matrix and linkage matrix, is computed. The higher value of the coefficient (closer to 1) authenticates that the distances between clusters/variables joined by the links through linkage function corroborate the distances between variables through dissimilarity function. The value of cophenetic correlation coefficient, $c=0.9582$ for the dendogram substantiates the closeness of linked variables/clusters in accordance with their actual distances in the input space.

In order to partition a set of variables into natural clusters, a measure of inconsistency is computed. The smallest value of this measure between two variables/clusters indicates that these variables/clusters are highly indistinguishable. Additionally, the inconsistency measure differentiates the clusters based on heights of the links joining them. The inconsistency measures for the links in cluster tree, Fig. 6, is presented in Table 4, second row and third row display the number of links at different levels and their inconsistency coefficients, respectively. Here, inconsistency coefficient ‘0’ corresponds to the links joining the pairs ${\text {NO}}_3$ (12) & F (15), Mg (6) & Cl (8), Ca (5) & As (16) and Na (9) & ${\text {SO}}_4$ (11) in Fig. 6, as there is no link joining the variables below them. The links joining these variables are also called leaf nodes.

**Figure 7:**
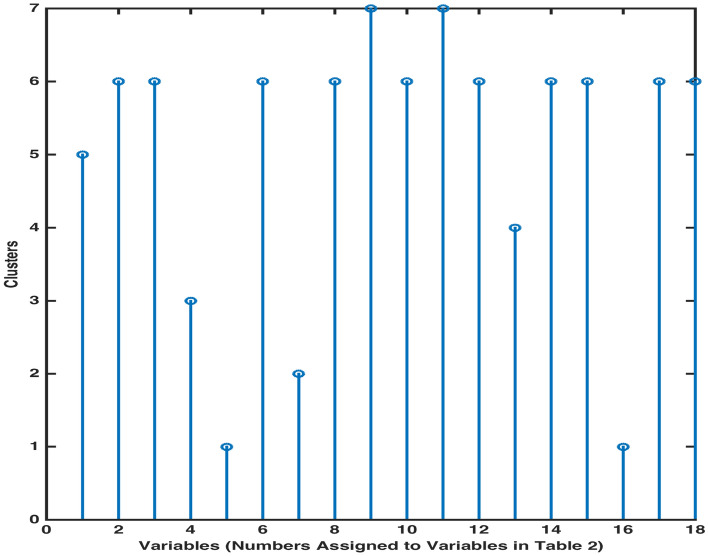
Stem diagram for clusters’ classification.

To determine an exact number of clusters for a set of 18 variables, a cuttoff inconsistency coefficient is chosen. This measure distinguishes the boundaries of clusters, each cluster comprising of similar characteristics’ variables. A cutoff inconsistency coefficient 0.7842 was considered to determine the boundaries of clusters in the dendogram. It assisted in allocating all variables to 7 clusters. To showcase this division, a stem diagram, Fig. 7, placing variables Na (9), ${\text {SO}}_4$ (11); pH (2), Turbidity (3), Mg (6), Cl (8), K (10), ${\text {NO}}_3$ (12), Fe (14), F (15), Total Coliforms (17), *E. Coli* (18); EC (1); TDS (13); $\text {HCO}_3$ (4); Hard (7); Ca (5), As (16) into clusters 7, 6, 5, 4, 3, 2, 1, respectively, is presented.

**Table 4:** Inconsistency measure for links in dendrogram.

Sr.	1	2	3	4	5	6	7	8	9	10	11	12	13	14	15	16	17
Number of links	1	2	2	2	2	2	1	2	1	3	3	1	3	2	2	2	2
Inconsistency coefficient	0	0.71	0.71	0.71	0.71	0.71	0	0.71	0	0.89	1.15	0	0.78	0.71	0.71	0.71	0.71

## Discussion

A comparison of Hierarchical Clustering (HC) and Neural Network Clustering (NNC) was conducted for clusters’ division of the data. HC provides a rigorous computing mechanism to segregate the components of a system based on explicit characteristic counts of the predictor variables. The gained partitioning was further refined using cophenetic coefficient and inconsistency coefficient. In analogy, the method of NNC was used to strongly indicate the visual discrimination and similarities of the predictor variables through the training plots.

A Self-Organizing Map (SOM)-Neural Network Clustering was adopted to group similar characteristic water variables into clusters. A Batch Learning Algorithm was used to identify classification of variables according to their grouping in an input space, 16 locations with 18 parametric values. Three aspects of SOM; Sample Hits, Neighbouring Weight Distances, Weight Planes were considered to highlight water components’ disposition and consolidation. The visual of sample hits, Fig. 3 demonstrates that two locations’ data is strongly correlated with respect to parametric elements, synonymous with a neuron marked ‘2’. This map also indicates that 16 samples locations can be categorized into 8 groups based on data conformity features. In addition to sample hits, dissimilarity of 16 locations’ vectors was determined through neighbouring distance of weighted inputs in Fig. 4. On account of about 6% of disparity among vectors in an input space, corresponding to dark red or black patches joining the weight vectors, only a very few locations’ samples will tend to show an outlying feature. A keen view of the data can detect anomalous values of four parameters in four different locations. To classify the highly correlated water variables, a weight plane visual Fig. 5 exhibiting weights (corresponding to each parameter) associated with neurons is obtained. Based on similar patterns of weight planes and identification of larger weights’ association with light color neurons, EC, $\text {HCO}_3$, Cl, Na, TDS, F; Turbidity, Ca, Hard, Fe; Mg, K; ${\text {SO}}_4$, ${\text {NO}}_3$; Coliforms, *E. Coli*; pH, As can be placed in 6 clusters.

An ML algorithm ‘clusterdata’ was used to obtain a cluster tree Fig. 6, displaying different levels containing clusters of water concentration variables. The co-application of dissimilarity function and linkage function facilitated the linking of a pair of clusters/ a pair of variable & a cluster based on the closest proximity distance between them. To verify whether linking of variables into clusters in the dendogram is an accurate representation of variables’ similarity or difference in a real system, a cophenetic coefficient was computed. The higher value of cophenetic measure affirms clustering efficiency of dendrogram to partition the data based on dissimilarity features. A cut off inconsistency coefficient, a measure to compare heights of links in dendrogram, served to provide an exact number of clusters encompassing 18 variables. The classification of variables based on their links’ heights into 7 clusters is demonstrated in Fig. 7; similar link lengths’ variables are placed in similar clusters.

It is observed that an SOM method of Neural Network Clustering (NNC) highlights some immediate features of a system such as from among 16 locations’ samples, 4 samples are quite distinct and remaining 12 samples are resembling with unsubstantial component differences. Also, the number of parameters exceeding their normal range by a long margin in samples from 16 locations was transmitted as 4, revealing the presence of four definite concentration parameters as outliers. However, this information is ambiguous and requires further inquiry methods to obtain precise locations or parameters with anomalous features. Another feature of SOM proclaimed of assembling 18 parameters into 6 clusters, based on their coincidental weight patterns. In contradiction to cursory information accumulation through sample hits and neighbouring weight distances, the information obtained through weight plane feature of SOM was specific and precise. On the other hand, Neural Network learning of the system has been compared with categorization information gathered through Hierarchical Clustering (HC). It is observed that clusters in dendrogram, obtained through cross-validating computing procedures of linkage and dissimilarity, accurately partition the data, with 44% comparability of HC with NNC. The reliability of HC to classify the variables into 7 clusters in contrast to 6 clusters-distribution through NNC has also been proven through a higher value of cophenetic coefficient. It has been emphasized through application of both methods that robust visual inference obtained through NNC can be interpreted and combined with rigorous computing outcome of HC to create an accurate segmentation of input vectors/predictor variables. It was observed that subtle hued association of water elements to neurons in NNC strongly indicated of the correlation between water concentration variables. However, a precise number of elements, sharing characteristic similarity, was provided by HC’s cophenetic coefficient. The synergic combination of optical reasoning and computing diagnostic approach is proclaimed to generate an accurate cluster divide. Presumably, the advantage of NNC to underline significant features of a huge system with moderate transparency is established as SOM scheme can accommodate a multivariable system of large number of input elements. However, the precision of dimension reduction and consolidation of similar features’ variables through complementing HC and NNC design outweigh inconclusive information gained through independent HC and NNC approach.

## Conclusion

A comparison of SOM-Neural Network Clustering (NNC) and Hierarchical Clustering (HC) is administered to assess computing dominance of either of the methods to classify a huge set of variables into clusters. An accurate clustering disposition can assist in scaling down a populous data without compromising essential knowledge of a system. The results obtained through comparative analysis are as vital as the inherent process that led to precise clustering conclusion. Primarily, the method of HC was applied on a water dataset to obtain a stringent computing mechanism to segregate its components using numeric characteristics of the predictor variables. Secondly, to create a parallel visual narrative, the method of NNC was used to reveal pattern discrimination and similarities of the predictor variables through SOM training plots. The cluster division obtained by both methods displayed a discrepancy probability of 0.2. The elimination of clusters’ boundaries disparity in both methods and the partitioning accuracy were further ensured using cophenetic coefficient and inconsistency coefficient.

Specifically, the optical inference approach of NNC pointed to two locations with remarkable analogy, and presence of four out of range elements in 16 locations. Moreover, it is implied that singular conduct of 18 concentration variables over a period of time can be comparably inquired through aggregate influence of EC, $\text {HCO}_3$, Cl, Na, TDS, F; Turbidity, Ca, Hard, Fe; Mg, K; ${\text {SO}}_4$, ${\text {NO}}_3$; Coliforms, *E. Coli*; As, pH in batches 6, 5, 3, 2, 1, respectively, on a local water system. In addition to obtaining a cluster divide of the predictors (water components) and inputs (locations) through NNC, the accuracy of HC to categorize the predictors was established through cophenetic measure for dendrogram and stem estimate. It was demonstrated that cluster genesis through combined HC’s linkage and dissimilarity algorithms & NNC is more reliable than individual optical assessment of NNC, where varying a map size in SOM will alter the association of inputs’ weights to neurons, providing a new structure of the clusters. Moreover, HC-classification of predictors Na (9), ${\text {SO}}_4$ (11); pH (2), Turbidity (3), Mg (6), Cl (8), K (10), ${\text {NO}}_3$ (12), Fe (14), F (15), Total Coliforms (17), *E. Coli* (18); EC (1); TDS (13); $\text {HCO}_3$ (4); Hard (7); Ca (5), As (16) into clusters 7, 6, 5, 4, 3, 2, 1, respectively, was retrieved through the cut-off coefficient, 0.7842. The current analysis presents the facts, that establish an accuracy dominance of linked HC-NNC approach in clustering the elements with assembly-influence on a system over an independent HC and NNC execution.

## Data Availability

The data that supports the findings of this study is available from the corresponding author, Nazish Shahid, upon reasonable request.
